# The mental lexicon: A blueprint for the dictionaries of tomorrow?

**DOI:** 10.3389/frai.2022.1027392

**Published:** 2023-01-25

**Authors:** Michael Zock

**Affiliations:** Aix Marseille Université, CNRS, LIS, Marseille, France

**Keywords:** word finding, *tip of the tongue*, mental lexicon, brain, cognition

## Introduction

When speaking or writing, we encounter either of the two situations: one where everything flows like water in a river or waves in the sea (Kempen and Hoenkamp, 1987) and the other, less peaceful situation which is filled with unpleasant surprises, with word-finding problems being one of them. The *tip of the tongue* (ToT) is probably the most frustrating situation as the author feels that he knows but cannot show it. ToTs can be a real nuisance, as they cause disruptions ranging from short delays to complete silence^1^. Being lost for words, or, more precisely, being blocked somewhere along the road connecting *meaning* to *form*, the user struggles anxiously to capture the desired target word.

The above described situation (*automatic activation* vs. *deliberate search*, i.e., navigation) resembles, to some extent, what Kahneman (2011) describes in *Thinking Fast and Slow*^2^. Yet, our main problem is not processing speed, which is more of a side effect, though one with serious consequences. The main issue is retrieval or the author's inability to fully activate the target word. To prevent this from happening or to overcome it, we need tools to facilitate word finding.

This is where lexical resources come into play, with dictionaries being one of them. Unfortunately, most dictionaries are not useful for this goal^3^. While they were designed with the reader in mind they support access to meaning given some input (word form), they cannot do the contrary: take a given meaning and reveal the desired target form (output). The problem is that we do not know how to describe meanings, even though they are the most natural starting point^4^. In the following paragraphs, we review what current dictionaries can do and how they can be improved.

Lexicographers have conceived many kinds of dictionaries (Hanks, 2012)^5^. Yet, despite the great variety, there are only two kinds of lexicon: the internal lexicon and the external lexicon. The first one is the mental lexicon (ML), the internal lexicon, the one we carry with us every day, our brain, or, more precisely, part of it. The latter one refers to the paper- and electronic-version of dictionaries that we are all familiar with. In each one of them, data are “stored” and represented differently. Hence, words in books, computers, and the human mind are not quite the same.

Since we are all familiar with external dictionaries (EDs), let me start with them. Both paper- and electronic dictionaries are databases storing word forms (lemma) and word-specific information (meaning, spelling, grammar, usage, etc.). Their number of entries far outnumbers that of our ML. The stored information is reliable and, in principle, accessible at any time. Finally, given some input, EDs can reveal all the information they have for a given entry, such as all the synonyms or otherwise related words.

On the downside, dictionaries are static, not always fault tolerant, and often insensitive to factors like frequency and language level. Also, while being rich in terms of the number of entries compared to the ML, they are poor in terms of cross-references and encyclopedic links. Of course, these shortcomings are more pronounced for paper dictionaries than for their electronic counterparts, which are much more flexible and performant in terms of fault tolerance and speed.

The situation is completely different for the mental lexicon. First, it is personal and comes before all other forms of dictionaries. More importantly, it has a number of features (power, flexibility, etc.) that make it unique and exceptionally powerful:

- Network and topology: All “words” are connected in various ways (encyclopedic-, episodic-, and lexical relations). This richness of links (density, variety, etc.), the size of the average path between any two nodes, and the type of connectivity (hubs) have important consequences for cognition in general (brainstorming, problem solving, and subliminal communication) and word-finding in particular. All words can be reached from anywhere *via* a small number of hops (small-world effect, Steyvers and Tenenbaum, 2005). Being connected to many other words (associations, co-occurrences, and so on), every target can be accessed *via* various routes. Last, every word evokes not only its core meaning but a whole world^6^.

- Relative importance: Words and connections have *weights* (expressing, for example, frequency, which may affect accessibility).
- Dynamism: The network is *self-organizing* and *continuously evolving*: weights change with the topic; entries are added; incomplete or faulty ones (gender) are corrected, and so on.
- Multi-purpose: The same brain takes care of different tasks (analysis/synthesis, translation, and more).
- Speed: The normal speaking rate of an average person in the US is about 2.5 words per second reading is twice as fast, and a Spanish soccer reporter speaks even faster. These are performances no dictionary can match, which is notable, as words are not stored holistically.
- Decomposition and gradual synthesis: In contrast to dictionaries, words are not readymade products stored in the brain. They result from a process comprising various steps that take place over time. Words can also be seen as a series of patterns distributed across various layers, each dedicated to a specific task (activation of meaning, form, or sound). For word production to take place, all patterns must be activated. While onlookers may have the impression that words are located and retrieved on the fly (single-step process), they are mistaken. Words are assembled, activated, or synthesized over time (Indefrey and Levelt, 2004). In sum, the brain is more of a word factory than a database.

To illustrate this last claim, let us present one of the major models in a nutshell (Levelt et al., 1999). Given some input (visual or else: idea, concept), the system performs the following three operations (meaning, form, and sound) to produce a spoken form^7^:

A. Activation of some *conceptual fragments* (dangerous semiaquatic animal), a broad *category* (“reptile”), an *image* (“
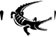
”), or an *idea*/*concept* (“logo of the Lacoste brand”).
B. Activation of the corresponding *lemma*: < “alligator” >/ < “caiman” > / < “crocodile” >, which, at this stage, still lacks the phonological form.
C. Activation of the concrete spoken or written form, i.e., *lexeme*: /ælɪgeɪtə/ vs. /reptaɪl/.

While ML is smaller in size and less reliable than EDs in terms of precision, it is much faster and more flexible than its competitors. This is due to the high number of vital connections for wordfinding. Given the importance of these qualities, one may wonder whether we could not use the ML as a model for the dictionaries of tomorrow^8^. Unfortunately, the answer is no, as this would imply that we are able to mimic the human brain, which is not possible for the following reasons:

a) The information needed to produce a word is distributed,
b) Knowledge is represented sub-symbolically; hence, it is indecipherable by humans,
c) The brain is in constant movement, affecting cognitive states, hence the availability of a given word.

While EDs do not have the qualities observed in the brain, their coverage is more extensive data are available anytime/anywhere, and the output is reliable and exhaustive. Given this, the question should be, which features can we choose from, the ML or the EDs, to get the best of both worlds? The answer seems to lie in the satisfaction of two constraints, (a) the building of *brain-compatible software*, that is, software complying with certain features of the human brain^9^, and (b) the building of a common language ensuring both the user and the resource (dictionary) understand each other. This implies that the system understands the user's goals^10^ and the user of the system. To this end, input/output must be holistic and symbolic.

In fact, this is already partially the case for various resources (see Table 1). Yet, the points where all of them could be improved are coverage, indexing, and integration. For promising work concerning this last point, integration, see the work devoted to multiplex networks (Lee et al., 2014; Castro et al., 2020).

**Table 1:** Potential dialogues between a human and a lexical resource based on the user's momentary knowledge state.

**Input: seed + (goal)**	**Output: unavailable, yet, desired target word**	**Type of search**	**Type of resource**
big size (*signifier*), (i.e., word form)	huge	semantic search: *definition*	Reverse dictionary: www.onelook.com
stab (*synonym*)	thrust	semantic search: *synonym*	Semantic network: https://wordnet.princeton.edu
bread (*topic: food*)	pastry	semantic search: *topical category*	Thesaurus: http://www.roget.org
hot (*opposite*) hand (*member*)	cold; finger	*semantically related word*	Semantic network: https://wordnet.princeton.edu
cat (*more general*) dog (*more specific*)	animal; Labrador	*hierarchically related word*	Semantic network: https://wordnet.princeton.edu
coffee (*association*)	strong; Starbucks	*association, co-occurrence*	Association thesaurus: http://rali.iro.umontreal.ca/rali/?q=en/USF-FAN
China (*encyclopedia*)	wall	*encyclopedic* knowledge	Knowledge graphs: https://babelnet.org, https://conceptnet.io
9/11 (*episodic knowledge*)	New York	*episodic* knowledge	social media (web), Yahoo
Australia (*capital*)	Canberra	*named entities*	http://nlp.cs.nyu.edu/ene/
publish (*rime*)	perish	search by *sound*	Rhyme dictionary: www.onelook.com; https://www.rhymezone.com

If we consider ML to be more of a huge knowledge graph encoding encyclopedic and episodic knowledge rather than a simple association network (Deyne and Storms, 2015), then it is good to know that there are already a lot of resources (https://www.dbpedia.org, https://babelnet.org/news, https://conceptnet.io, to name just a few). Although somehow strange, they have never seriously been considered as candidates to facilitate word finding. Why not give it a try?

## Author contributions

The author confirms being the sole contributor of this work and has approved it for publication.

## Footnotes

^1^The ToT refers to a state where the author knows a word and recalls part of it but is unable to recall its full form. It is by far the most widely studied and probably the most common word-finding problem (Schwartz, 2001). It occurs in all spoken languages and even in other modalities, for example, sign languages (Thompson et al., 2005), the *tip of the finger*, or the writing of Chinese characters, the *tip of the pen* (Huang et al., 2021).

^2^It is highly likely that the mental operations in spontaneous word access (fast) and deliberate search (slow) are not quite the same. Even if the second case also occurs in our brain without access to an external resource, the strategies used probably have much in common with offline processing, where people choose a specific lexical resource (see footnote ^5^) to “find” the word they are looking for.

^3^One must admit, though, that there are some resources to facilitate word finding (Zock and Biemann, 2020).

^4^This last criticism holds for dictionaries but not for thesauri (Roget, 1852) or “logographic,” i.e., morpho-syllabic writing systems (Chinese, Japanese). While the former lists words only in alphabetical order, the latter groups them by topical categories (food, instrument, etc.) or the character's elements (keys/radicals + number of strokes), two very useful indexing schemes for meaning-based search.

^5^For example, (A) *analogical-*, bilingual-, *synonym-, collocation-*, and reverse dictionaries; (B) *network-based lexical resources*: WordNet, Framenet, BabelNet, VerbNet, MindNet, and HowNet; (C) *web-based resources*: http://www.lexfn.com, OneLook, http://onelook.com.

^6^This feature casts serious doubts on the psycholinguistic reality of the “mental LEXICON” as a special module in our brain. For a similar point, though based on a different line of reasoning, please read the paper by Elman (2004). The point I care about here is the following: since every word is connected to all other words it typically occurs with, every word encodes a small part of world knowledge (Paris -- → capital, -- → France, -- → culture). There is no good reason to believe that ordinary language users (those who are not concerned by grammar or linguistic theory) make a distinction between word and world knowledge. They learned, maintained, and extended their lexical competency by seeing words in context, that is, connected to other words. This is how they learn something (facts, feelings) about words and the world. Notably, words in (con)texts provide naturally a rich set of indexing schemes able to support (creative) thinking, storage (encoding and memorization), and retrieval (word finding).

^7^Levelt's model is quite a bit more sophisticated. It requires the following six steps: (1) *conceptual preparation* -- → lexical concept; (2) *lexical selection* (abstract word) -- → lemma; (3) *morphological encoding* -- → morpheme; (4) *phonological encoding* (syllabification) -- → phonological word; (5) *phonetic encoding* -- → phonetic gestural code; and (6) *articulation* -- → sound wave. Notably, it assumes two knowledge bases: the *mental lexicon* for lemma retrieval and the *syllabary* for phonetic encoding.

^8^Please note that the author discusses here *deliberate search* (slow processing) and not the fast processing (stepwise *activation*) described in minute detail in Levelt's model.

^9^Translation of internal, subsymbolic mental operations into external symbolic actions. For example, information-spreading -- → navigation in an “association network”; variable cognitive states -- → variable inputs.

^10^This also requires that the user provides as input not only the word that comes to his/her mind (expressing his momentary cognitive state) when he/she cannot access (or, activate) the target word but also his/her goal, as otherwise, the receiver (resource) will not know what to do. This goal is usually dealt with implicitly by choosing a specific lexical resource (for example, a synonym dictionary rather than a thesaurus). It should also be noted that the user signals *via* his/her input both the nature of his/her knowledge gap and its location within the whole process of word production (meaning, form, and sound). This being so, he/she expects the system to produce either the word he is looking for or a set of words containing the target from which he will choose.
